# FastSpar: rapid and scalable correlation estimation for compositional data

**DOI:** 10.1093/bioinformatics/bty734

**Published:** 2018-08-29

**Authors:** Stephen C Watts, Scott C Ritchie, Michael Inouye, Kathryn E Holt

**Affiliations:** 1 Department of Biochemistry and Molecular Biology, Bio21 Molecular Science and Biotechnology Institute, The University of Melbourne, Parkville, Australia; 2 Systems Genomics Lab, Baker Heart & Diabetes Institute, Melbourne, Australia; 3 Department of Public Health and Primary Care, University of Cambridge, Cambridge, UK; 4 Department of Clinical Pathology and School of BioSciences, The University of Melbourne, Parkville, Australia

## Abstract

**Summary:**

A common goal of microbiome studies is the elucidation of community composition and member interactions using counts of taxonomic units extracted from sequence data. Inference of interaction networks from sparse and compositional data requires specialized statistical approaches. A popular solution is SparCC, however its performance limits the calculation of interaction networks for very high-dimensional datasets. Here we introduce FastSpar, an efficient and parallelizable implementation of the SparCC algorithm which rapidly infers correlation networks and calculates *P*-values using an unbiased estimator. We further demonstrate that FastSpar reduces network inference wall time by 2–3 orders of magnitude compared to SparCC.

**Availability and implementation:**

FastSpar source code, precompiled binaries and platform packages are freely available on GitHub: github.com/scwatts/FastSpar

**Supplementary information:**

Supplementary data are available at *Bioinformatics* online.

## 1 Introduction

Microbiome analysis, which aims to assay the bacterial communities present in a given sample set, is important in many fields spanning from human health to agriculture and environmental ecology. The current standard for investigating bacterial community composition is to deep sequence the total genomic DNA or the bacterial 16S rRNA gene and analyze the genetic diversity and abundance within each sample. Unique sequences or sequence clusters are taken to represent operational taxonomic units (OTUs) present in the original sample, and the frequencies of these across samples are summarized in the form of an OTU table (Ju and Zhang, 2015). In many studies, this data is then exploited to construct correlation networks of OTUs spanning sample sets, which can be used to infer or approximate interactions between taxa (He *et al.*, 2017; Nakatsu *et al.*, 2015).

The calculation of OTU correlation values is complicated by the sparse and compositional nature of the data. OTU counts are typically normalized by dividing each observation within a sample by the total count for that sample, giving a measure of relative abundance. However this transformation introduces dependencies between normalized sample observations, such that calculating simple correlations from the resulting values is not statistically valid (Aitchison, 1982). To perform robust and unbiased statistical analysis of sparse compositional data, it is generally first transformed from the simplex to Euclidean real space.

Returning compositional OTU data back to Euclidean real space can be achieved by taking the log ratio of OTU fractions. Utilizing log-ratios restores independence for each OTU and allows components to take on a positive or negative value. Building upon the use of log ratios, Friedman and Alm (2012) articulate an important and robust algorithm, SparCC, to estimate the linear Pearson Correlation between OTUs from variances of log ratios. Given that correlations cannot be calculated directly from log ratio variances, SparCC estimates the correlation statistic by using log ratio variances to approximate the true OTU variance on the assumption that the number of strong correlates is small (Friedman and Alm, 2012).

A Python 2 implementation of the SparCC algorithm has been released by the authors with several ancillary scripts for *P*-value estimation. However, the performance of this implementation precludes analysis of large datasets such as those generated from longitudinal studies (Teo *et al.*, 2017). Further, the *P*-value estimator used by SparCC has been demonstrated to be biased and overestimate significance (Phipson and Smyth, 2010).

Here we present FastSpar, a fast and parallelizable implementation of the SparCC algorithm with an unbiased *P*-value estimator. We demonstrate that FastSpar produces equivalent OTU correlations as SparCC while greatly reducing run time and memory consumption on large datasets. We also show that FastSpar has superior performance to the unpublished re-implementations of SparCC available in the mothur and SpiecEasi packages (Supplementary Fig. S1).

## 2 Implementation

FastSpar is written in C++11, utilizing OpenBLAS and LAPACK via the Armadillo library (Sanderson and Curtin *et al.*, 2016; Dongarra *et al.*, 1992; Xianyi *et al.*, 2012). The GNU Scientific Library (GSL) provides functionality for OTU fraction estimation and threading support is delivered by OpenMP (Dagum and Menon, 1998). In place of the *P*-value estimator used in SparCC, we utilized an estimator which corrects *P*-value understatement by considering the possibility of recalling repetitious permutations or original data during testing (Phipson and Smyth, 2010).

## 3 Results

### 3.1 Algorithm fidelity

To demonstrate that FastSpar produces equivalent correlations as SparCC, correlation networks were constructed by both programs using random subsets of an OTU table generated from the American Gut Project 16S rRNA sequence data (www.americangut.org), comprising a total of 6068 OTUs and 7523 samples. For each OTU pair, the mean correlation values calculated across 20 replicate runs were near identical for FastSpar and SparCC (Supplementary Figs S2 and S3). The observed OTU correlations calculated by SparCC and FastSpar are not reproduced exactly as there is a degree of randomness in the underlying algorithm. Specifically, OTU fractions are estimated by drawing from a Dirichlet probability distribution (parameterized using sample OTU counts with pseudocounts applied) and are therefore non-deterministic. Hence replicate runs of either program on the same input table produce similar but non-identical results (Supplementary Fig. S2A and B). To allow direct comparison of the algorithms, OTU fractions were pre-computed and provided as an additional input to both SparCC and FastSpar [note that the behaviour of the pseudo-random number generators (PRNG) used by FastSpar (GSL) and SparCC (numpy) differ, thus seeding the PRNGs is insufficient to enable direct comparison]. When using the same pre-computed OTU fractions as input, FastSpar and SparCC returned identical results (Supplementary Fig. S2D). These comparisons can be reproduced by running the code at github.com/scwatts/fastspar_comparison.

### 3.2 Performance profiling

**Fig. 1.**
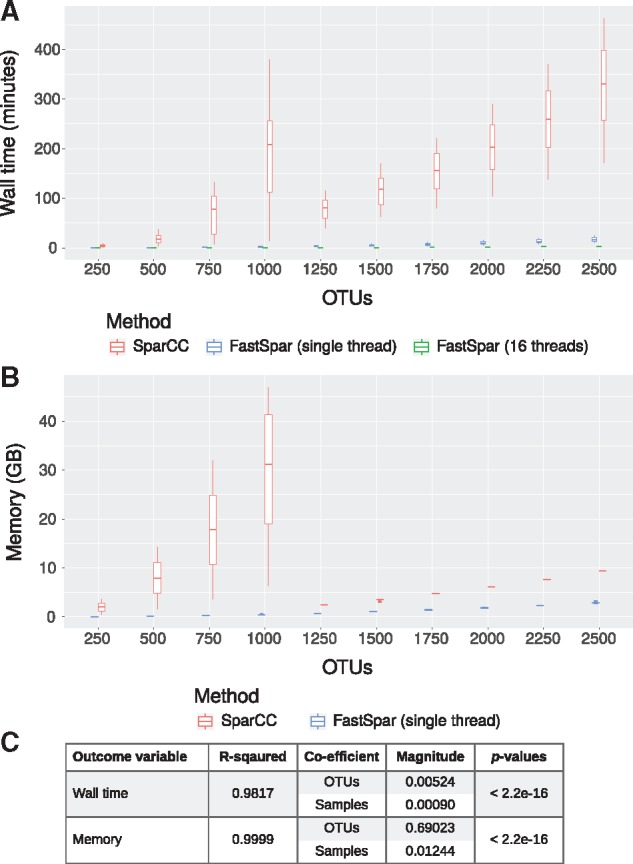
Performance profile of FastSpar and SparCC across random subsets of different sizes, extracted from the American Gut Project OTU table. (**A**) Wall time and (**B**) memory profiles were recorded using GNU time. (**C**) Linear models describing FastSpar (single thread) performance metrics with relation to input data dimensions

Performance was compared by running FastSpar and SparCC on random subsets of the American Gut Project OTU table (Fig. 1). Ten random subsets of each combination of sample sizes (*n* = 250, 500, …, 2500) and OTUs (*n* = 250, 500, …, 2500) were generated, and subjected to analysis using FastSpar (with and without threading) and SparCC. Wall time and memory usage was recorded using GNU time. The analysis was completed in an Ubuntu 17.04 (Zesty Zapus) chroot environment with the required software packages (Supplementary Table S1). Computation was performed with an Intel(R) Xeon(R) CPU E5-2630 @ 2.30GHz CPU and 62 GB RAM. The performance profiling can be reproduced by running the code at github.com/scwatts/fastspar_timed.

Using 16 threads, FastSpar was up to 821× faster than SparCC, (mean 221× faster; Fig. 1A). Using a single thread, FastSpar was up to 118× faster than SparCC (mean 32× faster; Fig. 1A). The memory usage of FastSpar was up to 116× less than SparCC (mean 26× less; Fig. 1B). Notably the memory performance of SparCC on datasets with more than 1000 OTUs improves considerably and is due to the conditional use of a more memory efficient calculation for the variation matrix (Fig. 1B). This conditional calculation appears to be beneficial for SparCC when analyzing datasets with 500 or fewer OTUs but causes a substantial performance degradation for datasets with 500–1000 OTUs (Supplementary Fig. S4).

As expected, both run time and memory principally scale with OTU number rather than sample number (Fig. 1C). For large datasets, it is therefore essential to perform pre-processing of the OTU table in order to reduce the number of OTUs prior to calculating correlations. This can be achieved primarily using two approaches: (i) filtering poorly represented OTUs, or (ii) distribution-based clustering such as that used in dbOTU3. The latter approach aims to reunite OTUs derived from sequencing error with the parent OTU by clustering OTUs based on nucleotide edit distance and count distribution (Preheim *et al.*, 2013). This has the advantage of retaining count information and thus improving statistical power. To simplify the workflow for large-scale correlation network analyses of microbiome data, FastSpar is packaged with an efficient C++11 implementation of dbOTU3 (github.com/scwatts/otudistclust) that has been optimized for analysis of large datasets by applying concurrency design patterns.

FastSpar provides a more robust and efficient method for inferring correlation networks from large microbiome datasets, which was previously intractable yet is likely to become commonplace in modern cohort studies.

## Funding

This work was supported by the National Health and Medical Research Council of Australia (Project #1062227, Fellowship #1061409 to K.E.H., Fellowship #1061435 to M.I. co-funded by the Australian Heart Foundation) and by the Australian Government Research Training Program (Scholarship to S.W and S.R.).

*Conflict of Interest*: none declared.

## Supplementary Material

Supplementary Data
