# Supplementary material for: FastSpar: rapid and scalable correlation estimation for compositional data
Source: Bioinformatics. 2018 Aug 29;35(6):1064–6. doi: 10.1093/bioinformatics/bty734 (PMC6419895; doi:10.1093/bioinformatics/bty734)
Supplement: Supplementary Data [file bty734_supplementary_data.pdf]

*Genetic and population analysis*

# FastSpar: Rapid and scalable correlation estimation for compositional data (supplementary data)

Stephen C. Watts[1*], Scott C. Ritchie[2,3,4], Michael Inouye[2,3,4], Kathryn E. Holt[1]

[1]Department of Biochemistry and Molecular Biology, Bio21 Molecular Science and Biotechnology Institute, The University of Melbourne, Parkville 3010, Victoria, Australia, [2]Systems Genomics Lab, Baker Heart & Diabetes Institute, Melbourne, Australia, [3]Department of Public Health and Primary Care, University of Cambridge, Cambridge CB1 8RN, United Kingdom, [4]Department of Clinical Pathology and School of BioSciences, The University of Melbourne, Parkville, VIC 3010, Australia

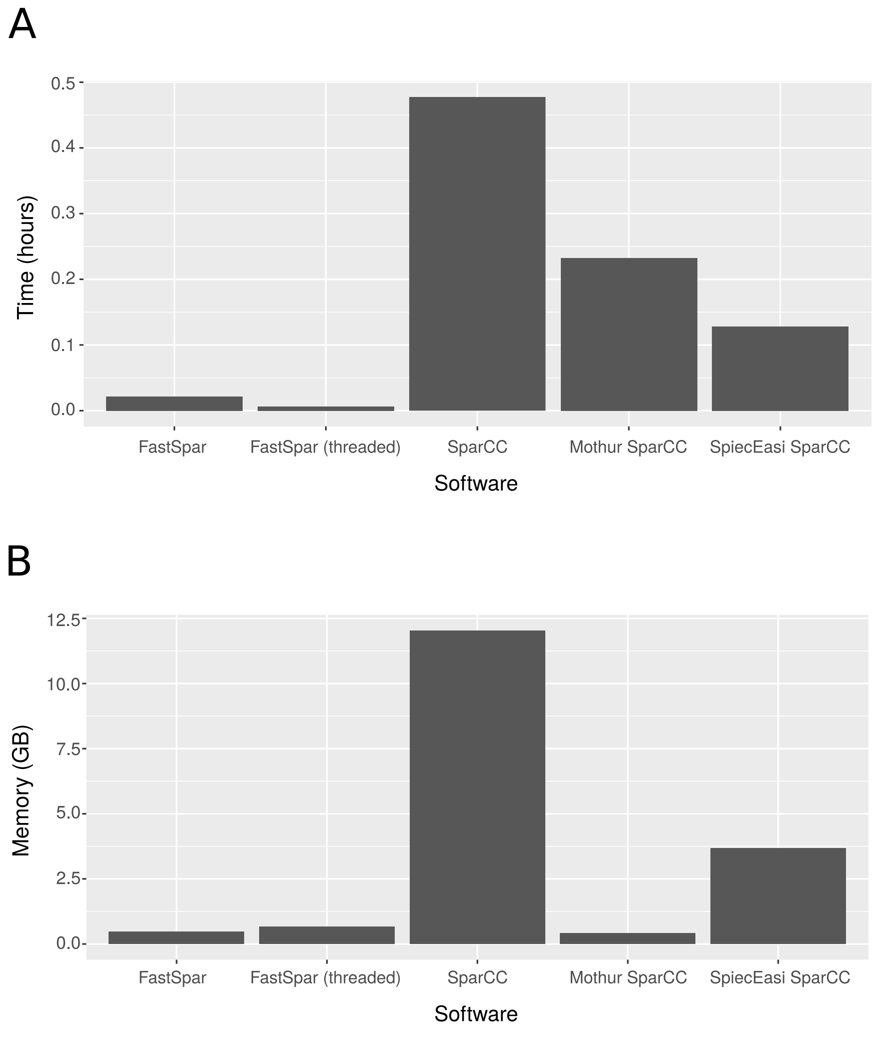


**Fig. S1.** Comparison of (**A**) time and (**B**) memory profiles for existing SparCC algorithm implementations. To perform profiling we used an OTU table composed of 500 samples and 1,000 OTUs randomly selected from the American Gut Project OTU table. Fastspar is demonstrated to have the shortest run time among the software packages. The Mother and SpiecEasi implementation of SparCC also show performance improvement with regards to both time and memory consumption.

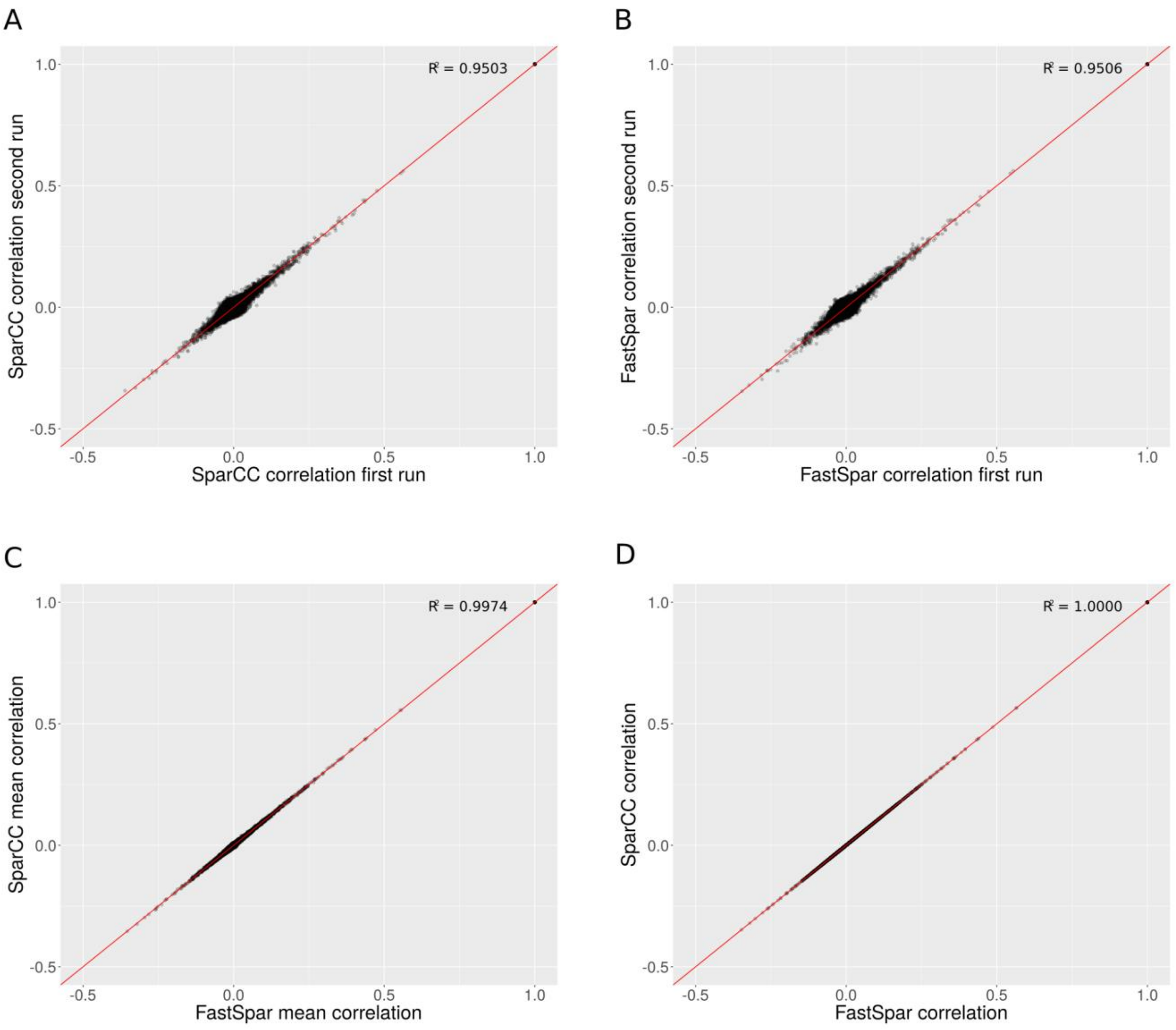


**Fig. S2.** Comparison of OTU correlation values estimated by FastSpar and SparCC. (**A-B**) Pairwise comparison of correlation values estimated across 20 replicate runs using the same implementation (**A**, SparCC or **B**, FastSpar). Note the algorithm is non-deterministic as OTU fractions are drawn from a probability distribution, hence variation of correlation values between replicates runs is observed with either implementation. (**C**) Pairwise comparison of mean estimates across 20 replicate runs, for SparCC vs FastSpar. Note that agreement between the mean estimates of the two implementations is greater than the agreement between replicate runs of the same implementation (panels **A-B**). (**D**) Direct comparison of correlation values generated by SparCC vs FastSpar using the same (i.e. non-random, pre-computed) OTU fractions, showing that FastSpar produces an identical result to SparCC.

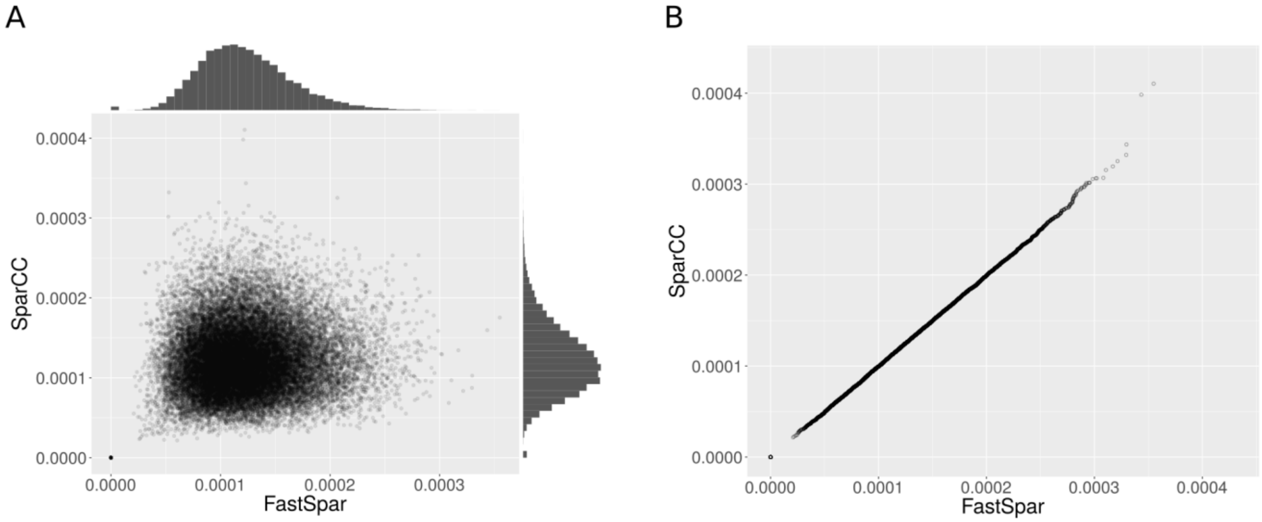


**Fig. S3.** (**A**) Distributions and (**B**) Q-Q plot of pairwise OTU correlation variance in 20 replicate runs of FastSpar and SparCC. OTU correlations were calculated for all pairs of 6,068 OTUs across 7,523 samples.

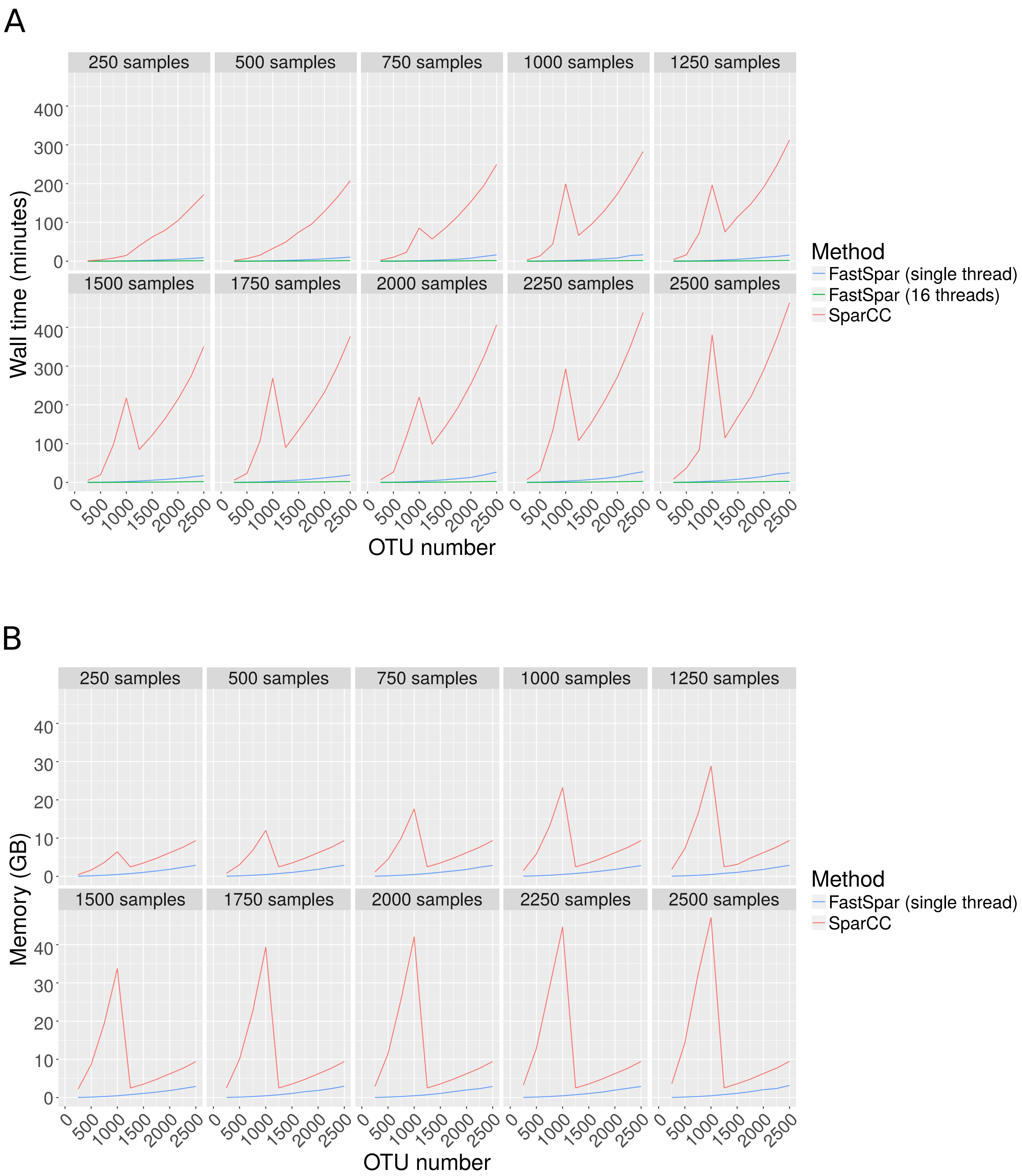


**Fig. S4.** Performance profile of (**A**) FastSpar and (**B**) SparCC for each individual random subset of the American Gut Project OTU table (full table contains 6,068 OTUs and 7,523 samples). Wall time and memory profiles recorded using GNU time.

**Table S1.** Software packages with version designations used for performance profiling and output comparison.

| Package | Version |
| --- | --- |
| autoconf | 2.69 |
| autoconf-archive | 20131101 |
| automake | 1.14.1 |
| build-essential | 12.1 |
| git | 2.7.4 |
| armadillo | 8.500.1 |
| libgsl-dev | 2.1 |
| libopenblas-dev | 0.2.18 |
| mercurial | 3.7.3 |
| python-numpy | 1.11.0 |
| python-pandas | 0.17.1 |
| python3-numpy | 1.11.0 |
| r-base-core | 3.2.3 |
| time | 1.7 |
